# Arsenic Trioxide Promotes Tumor Progression by Inducing the Formation of PGCCs and Embryonic Hemoglobin in Colon Cancer Cells

**DOI:** 10.3389/fonc.2021.720814

**Published:** 2021-10-05

**Authors:** Zugui Li, Minying Zheng, Hao Zhang, Xiaohui Yang, Linlin Fan, Fangmei Fu, Junjie Fu, Rui Niu, Man Yan, Shiwu Zhang

**Affiliations:** ^1^Department of Pathology, Tianjin Union Medical Center, Tianjin, China; ^2^Graduate School, Tianjin University of Traditional Chinese Medicine, Tianjin, China; ^3^Nankai University School of Medicine, Nankai University, Tianjin, China; ^4^Graduate School, Tianjin Medical University, Tianjin, China

**Keywords:** arsenic trioxide, colon cancer, polyploid tumor giant cells, cell fusion, epithelial-mesenchymal transition, erythroid cell

## Abstract

Arsenic trioxide (ATO) has been used to treat acute promyelocytic leukemia. However, it is not effective in treating solid tumors such as colorectal cancer. We have previously reported that polyploid giant cancer cells (PGCCs) exhibiting the characteristics of cancer stem cells can be generated by various inducers. In this study, ATO was used to induce the formation of PGCCs in LoVo and Hct116 colon cancer cell lines. The migration, invasion, and proliferation abilities of colon cancer cells with and without ATO treatment were assessed by wound-healing, transwell, and plate colony formation assays. The expression of epithelial to mesenchymal transition-related proteins and erythroid differentiation-related proteins in colon cancer cells was further evaluated by western blot and immunocytochemical assays. LoVo and Hct116 cells were transfected with a eukaryotic expression vector for green fluorescent protein (GFP), red fluorescent protein (RFP), H2B-GFP, and H2B-mCherry to study PGCCs formation *via* cell fusion. WB and ICC assays were performed to assess the expression of cell fusion-related proteins. MG132, small interfering RNA-glial cell missing 1 (GCM1), and chromatin immunoprecipitation-polymerase chain reaction assays were performed to study the role of GCM1/syncytin-1-mediated cell fusion. Clinically, the significance of cell fusion-related proteins and erythroid differentiation-related proteins expression in human colorectal cancer tissues was evaluated. Results of our study showed that ATO induced the formation of PGCCs, and the daughter cells derived from PGCCs gained a mesenchymal phenotype and exhibited strong migration, invasion, and proliferation abilities. PGCCs also produced embryonic hemoglobin-delta and -zeta with strong oxygen-binding ability and erythroid differentiation-related proteins after ATO treatment. In addition, cell fusion was observed during the formation of PGCCs, indicated by the presence of yellow fluorescence *via* the GCM1/syncytin-1 signaling pathway. Clinically, the expression of cell fusion-related and erythroid differentiation-related proteins gradually increased with the progression of human colorectal cancer tissues. In conclusion, ATO can promote tumor progression by inducing the formation of PGCCs *via* GCM1/syncytin-1-mediated cell fusion. PGCCs can produce daughter cells with high invasion and migration abilities and embryonic hemoglobin with strong oxygen binding ability, promoting survival of tumor cells in a hypoxic microenvironment.

## Introduction

Arsenic trioxide (ATO) has been widely used as a first-line agent in the treatment of acute promyelocytic leukemia (APL); however, it has not been found to be effective in the treatment of solid tumors, such as colorectal cancer, and its underlying mechanism of action is elusive (1, 2). Accumulating evidence suggests that among the bulk of leukemic cells, only a rare population of leukemia-initiating cells (LICs) propagates the disease. Recent reports have demonstrated that eradication of promyelocytic leukemia (PML)-retinoic acid receptor alpha by ATO treatment can clear LICs in APL in mice (3). ATO treatment can reduce PML protein expression levels and significantly decrease the number of quiescent LICs (4). Cancer stem cells (CSCs) were first described in acute myeloid leukemia (5) and solid tumors, such as colon cancer (6). Further, increasing evidence has shown that tumor growth and recurrence rely on the existence of CSCs, which are capable of self-renewal and differentiation into diverse tumor cell populations that form the bulk of tumors. CSCs are the key contributors to tumor initiation, heterogeneity, sustained growth, recurrence, metastasis, and therapeutic failure of tumors (7). In most cases, the traditional anti-tumor therapies commonly cause recurrence of solid tumors. One possible reason for this is that these treatments only target a large number of non-CSCs, but they do not eliminate a small number of CSCs (8). The significant activity of ATO in APL indicates that the targets of ATO in the treatment of APL may be CSCs rather than the common leukemia cancer cells (5, 9).

We have previously reported that polyploid giant cancer cells (PGCCs) are large cancer cells containing multiple copies of DNA that have cancer stem cell-like properties and are thought to generate cancer stem cell-like cells, contributing to cellular heterogeneity, chemoresistance, stemness, metastasis, and tumor progression in human solid tumor cells (10–15). In addition, a single PGCC can form spheroids and generate tumors, similar to CSCs. PGCCs can be induced from a wide variety of cancer cell lines using various inducers, including chemotherapy, radiotherapy, and hypoxia (10, 12, 16–18). PGCCs, resembling embryonic blastomeres, can also differentiate into all three germ layers *in vitro* (19, 20). PGCCs can generate regular-sized daughter cancer cells *via* asymmetric budding or bursting, and the tumors derived from PGCCs express embryonic stem cell markers, including OCT4, NANOG, CD44, and CD133 (10, 20). In addition, PGCC-derived tumors acquire a mesenchymal phenotype with increased expression of epithelial to mesenchymal transition (EMT) markers (21–24). Clinically, the number of PGCCs in tumor tissues is closely related to the pathological grade, clinical stage, distant metastasis, and resistance to radiotherapy and chemotherapy for malignant tumors (11, 16, 22). Furthermore, PGCCs can generate erythroid cells with increased expression of embryonic and fetal hemoglobin, which have a strong affinity for oxygen, promoting the survival of tumor cells in a hypoxic microenvironment (13, 14). The results of the current study showed that ATO induced the formation of PGCCs *via* glial cell missing 1 (GCM1)/syncytin-1 pathway-mediated cell fusion. During embryo development, GCM1/syncytin-1 pathway-mediated cell fusion is responsible for the fusion of cytotrophoblasts into syncytiotrophoblasts (25). Taken together, the poor effect of ATO on the treatment of colorectal cancers might be related to the formation of PGCCs, which can contribute to cellular heterogeneity, chemoresistance, stemness, metastasis, tumor progression, and survival in a hypoxic microenvironment by producing embryonic and fetal hemoglobin.

## Materials and Methods

### Cancer Cell Lines and Cultures

The human colon cancer cell lines LoVo and Hct116 were obtained from the American Type Culture Collection (ATCC; Manassas, VA, USA). LoVo and Hct116 cells were cultured in Roswell Park Memorial Institute-1640 medium (1×) (Gibco, Thermo Fisher Scientific, Suzhou, China) supplemented with 10% fetal bovine serum (FBS; Gibco, Life Technologies, New Zealand) and 1% penicillin-streptomycin (Gibco, Life Technologies, USA). Both cell lines were routinely incubated at 37°C in a humidified atmosphere containing 5% CO_2_.

### Formation of PGCCs and Spheroids From LoVo and Hct116 Cells After Induction by ATO

LoVo and Hct116 cells were cultured in complete 1640 medium in T25 flasks until they reached approximately 90% confluence. The cells were then cultured with 32 μM ATO (ATO was gifted by Professor GAO Yue, Department of Pharmaceutical Sciences, Beijing Institute of Radiation Medicine) for different times depending on their resistance to ATO. LoVo cells were treated with 32 μM ATO for 24 h, and Hct116 cells were cultured with 32 μM ATO for 48 h. The cells were then cultured in a regular complete medium after rinsing with phosphate-buffered saline (PBS; Gibco, Thermo Fisher Scientific, Suzhou, China). After treatment with ATO, the majority of the regular-sized cancer cells were killed, and the surviving cells with large size displayed obvious morphological changes. After 10–14 days of ATO removal, PGCCs generated daughter cells *via* asymmetric cell division. More and more daughter cells produced by PGCCs could attached to the surface of PGCCs and form spheroids in the medium. Finally, these spheroids either floated in the medium or attached to the wall of flask. After three ATO treatments, PGCCs with newly budding daughter cells and spheroids were collected for further analysis.

### Paraffin Embedding of Spheroids

The protocol of paraffin embedding of spheroids was described in our previous studies (12). In brief, spheroids from the medium were pipetted and centrifuged at 100g in the vials. The supernatant was removed and 70% ethanol was added to fix the spheroids. The samples were dehydrated and then the vials were infiltrated with acetone, absolute xylene and purified paraffin at 65°C for 15 min each. Finally, the spheroids were then embedded in paraffin and sectioned.

### Hematoxylin and Eosin Staining

LoVo and Hct116 cells with and without ATO treatment were cultured on slides for hematoxylin and eosin (H&E) staining. The cells were treated with ice-cold methyl alcohol for 30 min, rinsed with PBS, and then steeped in ultrapure water for a few seconds. The slides were stained with hematoxylin (Baso, Zhuhai, China) for approximately 1 min and then stained with eosin (Baso, Zhuhai, China) for 2 min. The cells were then dehydrated and mounted on coverslips, and the mean number of PGCCs was counted by randomly selecting five fields.

### PGCC Definition and Counting

PGCCs are defined as a subpopulation of tumor cells exhibiting large nuclei, which are at least three times larger than those of regular-sized diploid cancer cells. For each case, five microscopic fields were counted at 400× magnification, and the average number was calculated.

### Immunocytochemical Staining

LoVo and Hct116 cells with and without ATO treatment were cultured on slides in a 6-well plate with a complete medium until they reached 90% confluence. The cells were treated with ice-cold methyl alcohol for 30 min and rinsed with PBS. The cells were then blocked with an endogenous peroxidase inhibitor (Zhongshan Inc., Beijing, China) and subsequently with goat serum (Zhongshan Inc., Beijing, China) for 15 and 20 min, respectively, at room temperature. The primary antibodies (**Supplementary Table 1**) were incubated overnight at 4°C for 16 h. The cells were treated with secondary antibodies and horseradish peroxidase-labeled streptomycin (Zhongshan Inc.) for 20 and 15 min, respectively. They were then incubated with diaminobenzidine (Zhongshan Inc.) and counterstained with hematoxylin. The cells were rinsed twice with PBS and once with PBS with Tween-20 before incubation with each reagent (except serum) for 5 min.

### Wound-Healing Assay and Transwell Migration Assay

The migratory ability of LoVo and Hct116 cells was evaluated by wound-healing and transwell migration assays. Briefly, cells were incubated in a 6-well plate, and at least five horizontal lines were drawn at the back of the plate. The cells were then placed in each well and cultured overnight. Straight scratches were then made vertical to the line drawn on the back of the plate, and the wells were washed three times with PBS to remove excess cells. Cells were cultured in a serum-free culture medium at 37°C with 5% CO_2_ and observed at 0 h and 22 h. Thereafter, cell culture inserts (8 µm; Corning Inc.; 24-well plate) were used in the transwell migration assay. Cells (5 × 10^4^) in 200 µL medium (supplemented with 1% FBS) were seeded in the upper chamber, and 600 µL medium (supplemented with 20% FBS) was added to the lower chamber. After incubation for 24 h, the cell culture inserts were fixed with absolute methanol for 30 min and then stained with 0.1% crystal violet for 30 min.

### Transwell Invasion Assay

Transwell invasion assay was performed to assess the invasion ability of PGCCs with daughter cells. In brief, cell suspensions were prepared at 5 × 10^5^ cells per well and subsequently seeded in 200 μL medium without FBS onto inserts precoated with Matrigel basement membrane matrix (Corning). A growth medium with 20% FBS as the chemoattractant was added to the bottom chamber, and the plates were incubated for 12 h at 37°C in 5% CO_2_. After removing the medium without FBS and the upper chamber, the transwell inserts and invasive cells were fixed in methanol for 30 min and then stained using 0.1% crystal violet for 30 min. The number of cells that penetrated the membrane was counted in five fields.

### Plate Colony Formation Assay

Plate colony formation assay was performed to assess the proliferative ability of PGCCs. Briefly, 30, 60, and 120 cells were seeded into 6-well plates. The cells were cultured at 37°C with 5% CO_2_ for approximately 2 weeks. When macroscopic cell colonies appeared at the bottom of the plate, the cells were fixed with absolute methanol for 30 min and the cell colonies were stained with 0.1% crystal violet for 30 min. The number of cell colonies was counted using a microscope (clusters containing ≥50 cells were counted as a single colony). Colony formation efficiency was defined as the number of cells colonies/seeded cells.

### Establishing LoVo and Hct116 Cells With Stable Expression of Green Fluorescent Protein and Red Fluorescent Protein *via* Recombinant Lentiviral Vector

Recombinant lentiviral vectors encoding green fluorescent protein (GFP) and red fluorescent protein (RFP) genes were constructed using gene recombination technology. GFP and RFP genes were then introduced into human colon cancer cells to establish human colon cancer cell lines with stable expression of GFP and RFP (LoVo-GFP, LoVo-RFP, Hct116-GFP, and Hct116-RFP cells). Lentivirus negative control (NC) venom concentrate was synthesized by Gene-Pharma (Shanghai, China). Cells were selected using puromycin (Gene-Pharma, Shanghai, China). To visualize cell fusion in the formation of PGCC, human colon cancer cells with stable expression of GFP and RFP (LoVo and Hct116 cells) were co-cultured, i.e., LoVo-GFP cells were co-cultured with LoVo-RFP cells and Hct116-GFP cells were co-cultured with Hct116-RFP cells. PGCCs were generated from the co-cultured colon cancer cells expressing GFP and RFP after induction by ATO. Cell fusion of colon cancer cells was visualized as yellow fluorescence in the co-cultured colon cancer cells expressing GFP and RFP under a fluorescence microscope.

### Establishing LoVo and Hct116 Cells With Stable Expression of H2B-GFP and H2B-mCherry Proteins *via* Recombinant Lentiviral Vector

Recombinant lentiviral vectors encoding the human histone H2B-GFP and H2B-mCherry genes were constructed using gene recombination technology. Recombinant lentiviral vectors were synthesized by Gene-Pharma (Shanghai, China). Recombinant lentiviral vectors expressing H2B-GFP and H2B-mCherry were transfected into human colon cancer cells (LoVo and Hct116 cells) to obtain a stable cell line expressing H2B-GFP and H2B-mCherry. Cells were selected using puromycin (Gene-Pharma, Shanghai, China). To visualize nuclear fusion during the formation of PGCC, human colon cancer cells with stable expression of H2B-GFP and H2B-mCherry were co-cultured, i.e., LoVo-H2B-GFP cells were co-cultured with LoVo-H2B-mCherry cells and Hct116-H2B-GFP cells were co-cultured with Hct116-H2B-mCherry cells. PGCCs were generated from the co-cultured colon cancer cells expressing H2B-GFP and H2B-mCherry after induction by ATO. Nuclear fusion of colon cancer cells was visualized as yellow fluorescence in the co-cultured colon cancer cells expressing GFP and RFP under a fluorescence microscope.

### MG132 Inhibitor Treatment

After PGCCs were generated from colon cancer cells *via* cell fusion, PGCCs and control cancer cells were cultured in a 6-well plate until they reached 80% confluence, after which, 10 μmol/L MG132 (Target Molecular, Boston, USA) was added to each well for approximately 6 h, followed by lysis buffer for western blot (WB) analysis.

### Transient Small Interfering RNA Transfection

GCM1 was knocked down by transient small interfering RNA (siRNA) transfection. The siRNA oligonucleotides were synthesized by Gene-pharma (Shanghai, China), including three siRNA interference sequences (S1352, S815, and S201), one positive control sequence (GAPDH), one negative control sequence, and one mock control with only transfection reagents. Total protein extraction was performed by lysis in an ice-cold buffer for 30 min.

### Chromatin Immunoprecipitation Assay

Chromatin immunoprecipitation (ChIP) assay was performed to further verify the interaction between GCM1 and the *syncytin-1* gene in PGCC cells and control cancer cells using a commercial kit (Pierce Magnetic ChIP Kit 26157, Thermo Scientific). Briefly, cells were fixed with 1% formaldehyde to cross-link transcription factors into chromatin DNA and resuspended in a lysis buffer supplemented with a protease inhibitor mixture. The cells were then sonicated to shear chromatin DNA with lengths between 200 and 1000 base pairs. Immunoprecipitation was performed using GCM1 antibodies or control mouse immunoglobin G antibodies. After incubation with protein A-agarose/salmon sperm DNA, the antibody-protein-DNA-agarose complex was washed and harvested for subsequent reverse cross-linking. To assess the interaction between GCM1 and *syncytin-1*, the sheared DNA fragments from reverse cross-linking were extracted for further polymerase chain reaction (PCR) amplification using specific primers targeting the *syncytin-1* gene.

### Real-Time Fluorescent Quantitative PCR

The *Syncytin-1* primer was synthesized by Genewiz (Suzhou, China). The envelope primer sequences were chosen according to the Homo sapiens syncytin precursor mRNA (GenBank accession No. AF208161) as previously described (26). The *Syncytin-1* sense chain was 5’-CGT ACC CAT ACT CGC CTG GTA-3’ and the antisense chain was 5 ‘-GGT TTT GGG CCG AGA CCT-3’. Real-time fluorescent quantitative PCR was performed using a LightCycler480 instrument (Roche, Germany) to amplify a syncytin-1 specific sequence. PCR conditions were set according to the instructions provided by the SYBR Green Kit (Roche). The results were analyzed as described previously (27).

### WB Analysis

Hct116 and LoVo control cells without ATO treatment and PGCCs with daughter cells after ATO treatment were collected when approximately 80% confluence was reached. The cells were lysed on ice with approximately 100 μL of glacial radio-immunoprecipitation assay lysis buffer (Roche, Germany) for 30 min and then centrifuged at 14,000 rpm for 30 min at 4°C. The concentrations of the proteins were determined and separated by 10% sodium dodecyl sulfate-polyacrylamide gel. The protein bands were transferred onto polyvinylidene fluoride membranes (GE, USA). These membranes were blocked with 5% defatted milk (BD, USA) in 1 × Tris-buffered saline with 1% Tween-20 (Sigma, USA) for 2 h at room temperature. The membranes were then incubated with primary antibodies (**Supplementary Table 1**) at 4°C for 12 h. Subsequently, the membranes were incubated with secondary antibodies at room temperature for approximately 2 h. β-Actin was used as a protein-loading control. Protein expression was detected using a ChemiDoc imaging system (BioRad, USA).

### Tissue Samples

Paraffin-embedded human colorectal cancer tissue samples were obtained from the Department of Pathology, Tianjin Union Medical Center. None of the patients included in our study had been treated before surgical removal of the tumor. Pathologically, the 180 patients with colorectal cancer were divided into four groups including 49 patients with well-differentiated (group I), 42 patients with moderately differentiated (group II), 41 patients with poorly differentiated primary colorectal cancer (group III), and 48 patients with metastatic colorectal cancer (group IV). This study was approved by the hospital review board of Tianjin Union Medical Center. Furthermore, patient information was kept strictly confidential.

### Tissue Microarray

Formalin-fixed, paraffin-embedded tissues from these colorectal cancer samples were analyzed and stained with standard H&E, and tumor tissues without necrosis were chosen for tissue microarray construction with 1.5 mm cores (2.0 mm between cores). Two typical spots for each sample were chosen based on H&E staining.

### Immunohistochemical Staining

**Immunohistochemical** (IHC) staining was performed for all sections. Paraffin-embedded tissue sections were subjected to hyperthermal roasting at 70°C for 2 h, deparaffinized in xylene, and dehydrated using concentration-gradient ethanol solutions. Next, antigen retrieval was performed using a heated citrate buffer solution (Origene, Wuxi, China) in an autoclave at 100°C for 3 min. After blocking with endogenous peroxidase inhibitor and then with goat serum, the sections were incubated with primary antibodies (**Supplementary Table 1**) at 4°C for 16 h. The further processes were the same as those after incubation with primary antibodies for immunocytochemical (ICC) staining.

### IHC Scoring

The expression of cell fusion-related proteins (including GCM1, syncytin-1, and the human sodium-dependent neutral amino acid transporter type C-2 [ASCT-2]) and erythroid differentiation-related proteins (including hemoglobin-delta, hemoglobin-zeta, CD71, GATA-1, and GATA-2) in the tissue samples was evaluated and quantified based on the percentage of positive cells and staining intensity. The staining intensity was assessed as follows: 0, negative (no staining); 1, weakly positive (faint yellow staining); 2, moderately positive (brownish-yellow staining); and 3, strongly positive (brown staining). The number of positive cells was visually evaluated as follows: 0 (negative), <5% positive cells; 1 (weak), 6%–25% positive cells; 2 (moderate), 26%–50% positive cells; 3 (above moderate), 51%–75%; and 4 (strong), >76% positive cells. The staining index for each section was determined based on the sum of the staining intensity and positive cell scores, as described previously (22).

### Statistical Analysis

The statistical software SPSS 16.0 (IBM Corporation, Armonk, NY) was used to analyze all data in the present study. The Kruskal-Wallis test was used to evaluate the differences in cell fusion and erythroid differentiation-related protein expression between the four groups (Groups I–IV) of human colon tissues. All histogram data are presented as mean ± standard deviation. Statistical significance was set at *P* < 0.05.

## Results

### Formation of PGCCs From Colon Cancer Cells by the Induction of ATO

**Figure 1:**
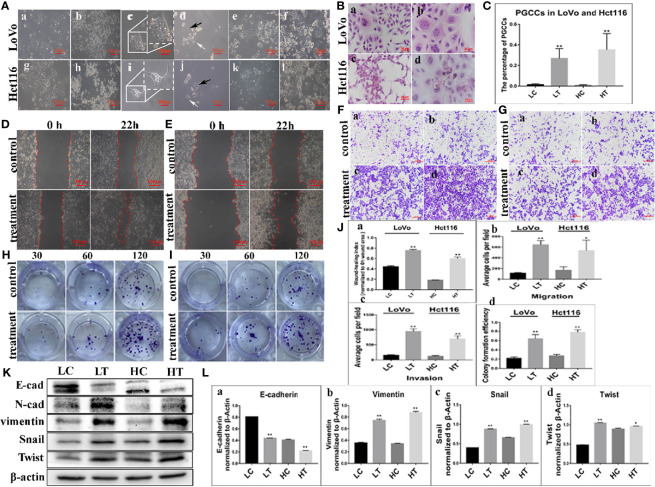
PGCCs with daughter cells after ATO induction. **(A)** PGCCs with daughter cells generated from colon cancer cells after ATO treatment (100×). **(a)** LoVo control cells. **(b)** LoVo after treatment for 24 h **(c)** Single PGCC survived after ATO treatment. **(d)** LoVo PGCCs (black arrow) generated daughter cells (white arrow) after recovery from ATO treatment. **(e)** After 10–14 days, more daughter cells derived from LoVo PGCCs were observed. **(f)** After treatment with ATO 3-5 times, more PGCCs and daughter cells were observed. **(g)** Hct116 control cells. **(h)** Hct116 after treatment for 48 h **(i)** Most regular-sized cells were killed, while Hct116 cells with enlarged nuclei survived (PGCCs). **(j)** Hct116 PGCCs (black arrow) generated daughter cells (white arrow) after recovery from ATO treatment. **(k)** After 10–14 days later, more daughter cells derived from Hct116 PGCCs were observed. **(l)** After treatment with ATO for 3–5 rounds, more PGCCs and daughter cells were observed. **(B)** H&E staining of Hct116 and LoVo cells before and after ATO treatment (200×). **(a)** H&E staining of the control LoVo cells. **(b)** More PGCCs appeared in LoVo cells after ATO treatment. **(c)** H&E staining of control Hct116 cells. **(d)** More PGCCs appeared in Hct116 cells after ATO treatment. **(C)** Comparison of the percentage of PGCCs in LoVo and Hct116 cells with and without ATO treatment. **(D)** Wound-healing assay in LoVo cells before and after ATO treatment at 0 h and 22 h (100×). **(E)** Wound-healing assay in Hct116 cells before and after ATO treatment at 0 h and 22 h (100×). **(F)** Transwell assays before and after ATO treatment in LoVo cells (100×). **(a)** Migration and **(b)** invasion of LoVo control cells. **(c)** Migration and **(d)** invasion of LoVo cells after ATO treatment. **(G)** Transwell assays before and after ATO treatment in Hct116 cells (100×). **(a)** Migration and **(b)** invasion of Hct116 control cells. **(c)** Migration and **(d)** invasion of Hct116 cells after ATO treatment. **(H)** Colony formation assay of 30, 60, and 120 cells before and after ATO treatment in LoVo cells. **(I)** Colony formation assay of 30, 60, and 120 cells before and after ATO treatment in Hct116 cells. **(J)** Column diagram showing the comparison of the abilities of Wound-healing **(a)** Migration **(b)**, invasion **(c)**, and the colony formation efficiency **(d)** in LoVo and Hct116 cells with and without ATO treatment (^*^P < 0.05,^**^P < 0.01). **(K)** Western blot analysis study of EMT-related protein expression including E-cadherin, vimentin, Snail, and Twist in LoVo and Hct116 cells with and without ATO treatment. **(L)** Column diagram showing the gray value differences of E-cadherin **(a)**, vimentin **(b)**, Snail **(c)**, and Twist **(d)** with and without ATO treatment (^*^P < 0.05,^**^P < 0.01). LC, LoVo control cells without ATO treatment; LT, LoVo cells with ATO treatment; HC, Hct116 control without ATO treatment; HT, Hct116 cells with ATO treatment.

LoVo cells (**Figure 1A-a**) and Hct116 cells (**Figure 1A-g**) were treated with 32 μM ATO for 24 h and 48 h, respectively. The regular-sized diploid cancer cells were killed (**Figure 1A-b**, **A-h**), while several scattered enlarged cells with multiple or giant nuclei (PGCCs) survived and displayed obvious morphological changes (**Figures 1A-c, A-i**). After 10–14 days, following recovery from ATO treatment, PGCCs started to generate daughter cells *via* asymmetric cell division (**Figures 1A-d, A-j**). When PGCCs with their daughter cells reached approximately 90% confluence after trypsinization (**Figures 1A-e, A-k**), ATO at the same concentration with the first treatment was used to treat the cells for the second time. After treatment with ATO three times, PGCCs with daughter cells and spheroids were observed (**Figures 1A-f, A-l**, **Figure S1A, B**) and collected for further analysis. The control cells and PGCCs with daughter cells after three ATO treatments were seeded onto a coverslip for H&E staining. The results of H&E staining showed that the percentage of PGCCs in cancer cells treated with ATO was higher than that in control cancer cells not treated with ATO (**Figures 1B-a–d**), and the differences were statistically significant for LoVo and Hct116 cells (**Figure 1C**).

### Increased Ability of Migration, Invasion, and Proliferation in PGCCs With Daughter Cells

The results of the wound-healing assay revealed a significant increase in the migration ability of LoVo and Hct116 cells after ATO treatment compared to control cancer cells without ATO treatment (**Figures 1D, E, J–a**). In addition, the transwell migration assay showed similar results (**Figure 1F, J–b**). Moreover, the transwell invasion assay indicated that cells that were treated with ATO had a stronger invasive ability than control cancer cells (**Figure 1G, J–c**). In addition, the plate colony formation assay demonstrated a higher proliferation capacity in cancer cells treated with ATO than in control cells not treated with ATO (**Figures 1H–J–d**). These results suggested that the migration, invasion, and proliferation abilities of LoVo and Hct116 cells were increased after treatment with ATO.

### Expression of EMT-Related Proteins in PGCCs With Daughter Cells After ATO Treatment

**Figure 2:**
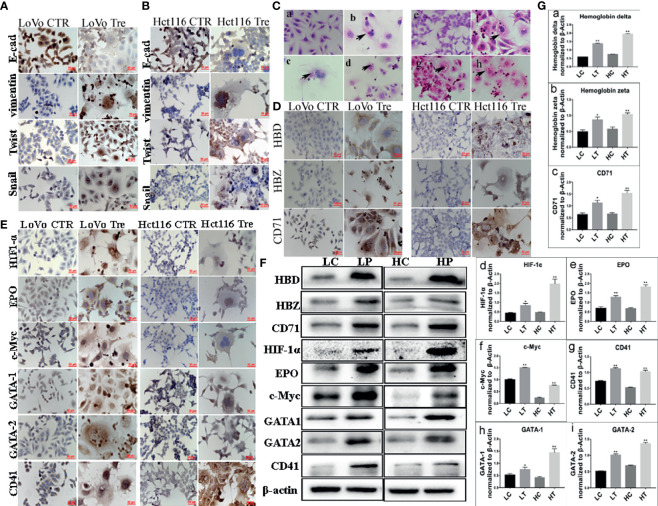
Expression of EMT-related proteins and erythroid differentiation-related proteins in LoVo and Hct116 cells with and without ATO treatment. **(A)** ICC staining in evaluating EMT-related proteins (including E-cadherin, Vimentin, Twist, and Snail) in LoVo and Hct116 cells before and after ATO treatment (200×). **(B)** Immunocytochemical (ICC) staining in evaluating EMT-related proteins in Hct116 cells (200×). **(a)** E-cadherin expression in cells without ATO treatment, **(b)** E-cadherin expression in cells with ATO treatment. **(c)** Vimentin expression in cells without ATO treatment, **(d)** Vimentin expression in cells with ATO treatment. **(e)** Twist expression in cells without ATO treatment, **(f)** Twist expression in cells with ATO treatment. **(g)** Snail expression in cells without ATO treatment, **(h)** Snail expression in cells with ATO treatment. **(C)** The results of H&E staining showed erythroid differentiation of LoVo and Hct116 cells after ATO treatment (black arrows head the erythroid cells, 400×). **(a)** Control LoVo cells. **(b–d)** Erythroid cells appeared in the cytoplasm of LoVo cells after ATO treatment. **(e)** Control Hct116 cells. **(f–h)** Erythroid cells appeared in the cytoplasm of Hct116 cells after ATO treatment. **(D)** ICC staining results of hemoglobin-delta, hemoglobin-zeta, and CD71 expression in LoVo and Hct116 cells before and after ATO treatment (200×). **(E)** ICC staining results of the expression of erythroid differentiation-related proteins (including HIF-1α, EPO, c-Myc, GATA-1, GATA-2, and CD41) in LoVo and Hct116 cells before and after ATO treatment (200×). **(F)** Western blot analysis results of erythroid differentiation-related protein expression (including HBD, HBZ, HIF-1α, EPO, c-Myc, GATA-1, GATA-2, and CD41) in LoVo and Hct116 cells with and without ATO treatment. **(G)** Column diagram showing the gray value differences of **(a)** HBD, **(b)** HBZ, **(c)** CD71, **(d)** HIF-1α, **(e)** EPO, **(f)** c-Myc, **(g)** CD41, **(h)** GATA-1, **(i)** GATA-2 in LoVo and Hct116 cells with and without ATO treatment (^*^P < 0.05,^**^P < 0.01). LC: LoVo control cells without ATO treatment LT, LoVo cells with ATO treatment; HC, Hct116 control without ATO treatment; HT, Hct116 cells with ATO treatment; HBD, hemoglobin-delta; HBZ, hemoglobin-zeta.

WB assays were performed to further assess the expression of EMT-related proteins, including E-cadherin, vimentin, Snail, and Twist, in LoVo and Hct116 cells with and without ATO treatment. The results revealed that the expression of E-cadherin was decreased (**Figures 1K, L-a**), while that of vimentin was increased in LoVo and Hct116 cells after ATO treatment compared to that of E-cadherin and vimentin in control cancer cells (**Figures 1K, L-b**). The EMT regulatory factors (N-cadherin, Twist, and Snail) were also more highly expressed in LoVo and Hct116 cells after treatment with ATO than in the control cancer cells without ATO treatment (**Figures 1K, L-c, L-d**). The results of ICC staining showed that the expression of E-cadherin was decreased, while that of vimentin, Snail, and Twist was markedly increased in LoVo and Hct116 cells after treatment with ATO compared to control cancer cells without ATO treatment (**Figures 2A, B**). The results of ICC and WB assays confirmed that EMT occurred after ATO treatment in LoVo and Hct116 cells, contributing to the strong abilities of migration, invasion, and proliferation.

### Expression of Erythroid Differentiation-Related Proteins in PGCCs With Daughter Cells

Our previous study indicated the ability of PGCCs with daughter cells to generate erythroid cells *in vitro* after cobalt chloride treatment (14, 20). In the present study, H&E staining of paraffin embedded spheroids derived from PGCCs indicated the presence of erythroid cells in the cytoplasm of PGCCs and the large nuclei of PGCCs (**Figures 2C-b–d, f–h**), and there were no erythroid cells in the control LoVo and Hct116 cells (**Figures 2C-a, C-e**). WB and ICC assays were performed to further confirm the expression of erythroid differentiation-related proteins in LoVo and Hct116 cells after ATO treatment. ICC results revealed that the expression of hemoglobin-delta and -zeta was increased, and that of CD71, a fetal cell marker transferrin receptor antigen, was also highly elevated in LoVo and Hct116 cells after ATO treatment compared to that in the control cells (**Figure 2D**). The ICC assay results indicated that the expression of erythroid differentiation-related proteins, including HIF-1α, EPO, c-Myc, GATA-1, and GATA-2, was increased in cancer cells treated with ATO (**Figure 2E**). In addition, ICC staining results showed that the expression of CD41, a specific marker of megakaryocytes, was markedly increased (**Figure 2E**). The results of WB assay further demonstrated that the expression of hemoglobin-delta (**Figures 2F, G-a**) and hemoglobin-zeta (**Figures 2F, G-b**), CD71 (**Figures 2F, G-c**), HIF-1α (**Figures 2F, G-d**), EPO (**Figures 2F, G-e**), c-Myc (**Figures 2F, G-f**), GATA-1(**Figures 2F, G-g**), GATA-2 (**Figures 2F, G-h**), and CD41 (**Figures 2F, G-i**) was increased in LoVo and Hct116 cells after ATO treatment. These results indicate the ability of PGCCs to generate erythroid cells with increased expression of embryonic and fetal hemoglobin.

### Establishing Stable Expression of GFP and RFP Genes and Stable Co-Expression of H2B-GFP and H2B-mCherry Genes *via* Recombinant Lentiviral Vector in LoVo and Hct116 Cells

**Figure 3:**
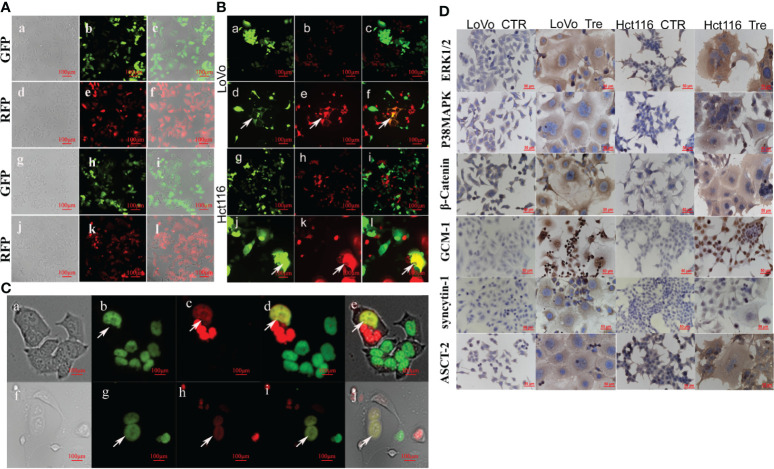
PGCCs formed *via* cell fusion and nuclear fusion in LoVo and Hct116 cells after ATO treatment observed by fluorescence microscopy. **(A)** Establishing LoVo and Hct116 cells with stable expression of GFP and RFP *via* recombinant lentiviral vector **(a)** Bright-field, **(b)** GFP fluorescence, and **(c)** merged image in LoVo cells without ATO treatment. **(d)** Bright-field, **(e)** RFP fluorescence, and **(f)** merged image in LoVo cells without ATO treatment. **(g)** Bright-field, **(h)** GFP fluorescence, and **(i)** merged image in Hct116 cells without ATO treatment. **(j)** Bright-field image, **(k)** RFP fluorescence (red) image, and **(l)** merged image in Hct116 cells without ATO treatment. **(B)** Cell fusion of PGCCs observed in co-cultured LoVo and Hct116 cells with stable expression of GFP and RFP after ATO treatment (white arrows head). **(a)** GFP fluorescence. **(b)** RFP fluorescence. **(c)** merged image in LoVo cells before ATO treatment. **(d)** PGCCs with GFP fluorescence. **(e)** PGCCs with RFP fluorescence. **(f)** Merged image (yellow) of PGCCs in LoVo cells after ATO treatment. **(g)** GFP fluorescence. **(h)** RFP fluorescence. **(i)** merged image in Hct116 cells before ATO treatment. **(j)** PGCCs with GFP fluorescence. **(k)** PGCCs with RFP fluorescence. **(l)** Merged image (yellow) of PGCCs in Hct116 cells with ATO treatment. **(C)** Nuclear fusion of PGCCs observed in co-cultured LoVo and Hct116 cells with stable expression of H2B-GFP and H2B-mCherry after ATO treatment (White arrows head). **(a)** Bright-field, **(b)** H2B-GFP fluorescent, **(c)** H2B-mCherry fluorescent, **(d)** Merged image (yellow) from H2B-GFP image and H2B-mCherry image, and **(e)** Merged image of bright-field, H2B-GFP and H2B-mCherry in LoVo cells after ATO treatment. **(f)** Bright-field, **(g)** H2B-GFP fluorescent, **(h)** H2B-mCherry fluorescent, **(i)** Merged image (yellow) from H2B-GFP and H2B-mCherry, and **(j)** Merged image from bright-field, H2B-GFP and H2B-mCherry in LoVo cells after ATO treatment. **(D)** ICC staining results of cell fusion-related protein, including ERK1/2, P38 MAPK, β-catenin, GCM1, syncytin-1, and ASCT-2, expression in LoVo and Hct116 cells before and after ATO treatment (200×). CTR, Control cancer cells; Tre, Cancer Cells with ATO treatment; LC, LoVo control cells without ATO treatment; LT, LoVo cells with ATO treatment, HC, Hct116 control without ATO treatment; HT, Hct116 cells with ATO treatment.

To visualize cell fusion and nuclear fusion in the formation of PGCC, LoVo and Hct116 cells with stable expression of GFP and RFP genes and stable co-expression of H2B-GFP and H2B-mCherry genes were constructed using recombinant lentiviral vectors. After selection with puromycin, most colon cancer cells stably expressed GFP and RFP proteins (**Figures 3A-a–f**), H2B-GFP, and H2B-mCherry (**Figures 3A-g–l**). Our results also showed that H2B-GFP and H2B-mCherry fusion proteins were highly specific for nuclear chromatin as no fluorescence was observed in the cytoplasm.

### Cell Fusion and Nuclear Fusion During the Formation of PGCCs After Induction by ATO

The control cancer cells with stable expression of GFP and RFP were co-cultured (the same amount of LoVo-GFP and LoVo-RFP cells as well as Hct116-GFP and Hct116-RFP cells were co-cultured; **Figures 3B-a–c, g–i**). After treatment with ATO, some PGCCs stably expressed GFP and RFP and displayed yellow when the two images were merged, indicating that the cells expressed both GFP and RFP (**Figures 3B-d–f, j–l**), which showed that ATO promoted the formation of PGCCs *via* cell fusion.

To further investigate whether DNA fusion occurred during the formation of PGCCs, control cancer cells with stable co-expression of H2B-GFP and H2B-mCherry were co-cultured (the same amount of LoVo-H2B-GFP and LoVo-H2B-mCherry cells as well as Hct116-H2B-GFP and Hct116-H2B-mCherry cells were co-cultured). There was no nuclear fusion in the co-cultured control cancer cells before ATO treatment. After ATO treatment, some PGCCs were yellow in LoVo (**Figures 3C-a–e**) and Hct116 cells (**Figures 3C-f–j**) when the two images were merged, indicating that the nuclei of PGCCs co-expressed both GFP and mCherry. These results showed that nuclear fusion occurred during the formation of PGCCs after treatment with ATO.

### Expression of Cell Fusion-Related Proteins in PGCCs With Daughter Cells

**Figure 4:**
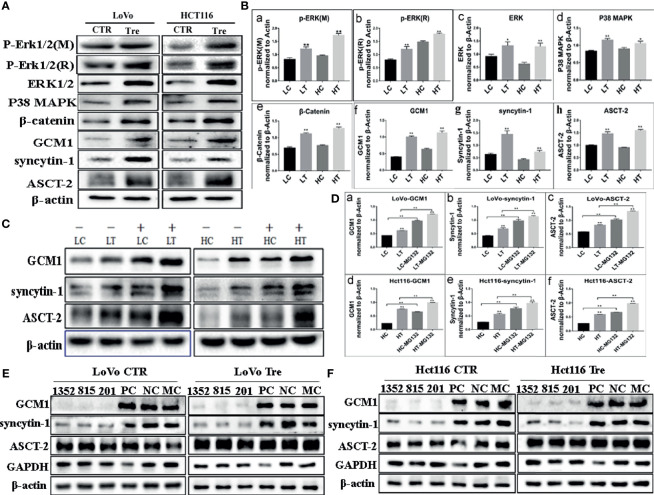
Expression of cell fusion related proteins in LoVo and Hct116 cells with and without ATO treatment. **(A)** Western blot analysis of cell fusion-related protein expression in the LoVo and Hct116 cells with and without ATO treatment. **(B)** Column diagram showing the gray value differences of WB of cell fusion-related proteins, including **(a)** p-ERK1/2 (mouse), **(b)** p-ERK1/2 (rabbit), **(c)** ERK1/2, **(d)** P38 MAPK, **(e)** β-catenin, **(f)** GCM1, **(g)** syncytin-1, and **(h)** ASCT-2, in LoVo and Hct116 cells with and without ATO treatment (^*^P < 0.05,^**^P < 0.01). **(C)** Western blot analysis of the expression of GCM1, syncytin-1, and ASCT-2 after MG132 treatment in the LoVo and Hct116 cells with and without ATO treatment. **(D)** A column diagram showing the gray value differences of protein expression after MG132 treatment in LoVo and Hct116 cells with and without ATO treatment (^*^P < 0.05,^**^P < 0.01). **(a)** GCM1 in LoVo cells, **(b)** syncytin-1 in LoVo cells, **(c)** ASCT-2 in LoVo cells, **(d)** GCM1 in Hct116 cells, **(e)** syncytin-1 in Hct116 cells, and **(f)** ASCT-2 in Hct116 cells. **(E)** Western blot analysis of total protein expression of GCM1, syncytin-1, and ASCT-2 in LoVo cells with and without ATO treatment after siRNA- GCM11352, 815, 201, siRNA control, and negative control transfection. **(F)** Western blot analysis of total protein expression of GCM1, syncytin-1, and ASCT-2 in Hct116 cells with and without ATO treatment after siRNA-GCM1 1352, 815, 201, siRNA positive control and negative control transfection. LC, LoVo control cells without ATO treatment; LT, LoVo cells with ATO treatment; HC, Hct116 control without ATO treatment; HT, Hct116 cells with ATO treatment; PC, Positive control; NC, Negative control; MC, Mock control.

To investigate the molecular mechanism involved in cell fusion, the expression of cell fusion-related proteins was evaluated by ICC and WB assays in LoVo and Hct116 cells with and without ATO treatment. ICC assay results indicated that the expression of cell fusion-related proteins, including ERK1/2, P38 MAPK, β-catenin, GCM1, syncytin-1, and ASCT-2, was increased in LoVo and Hct116 cells after ATO treatment compared to that in control cells (**Figure 3D**). WB assays also confirmed that the expression of cell fusion-related proteins, including p-ERK1/2(mouse), p-ERK1/2(rabbit), ERK1/2, P38 MAPK, β-catenin, GCM1, syncytin-1, and ASCT-2, increased compared to that in control cancer cells (**Figure 4A**), and the differences were statistically significant (**Figure 4B**).

**Figure 5:**
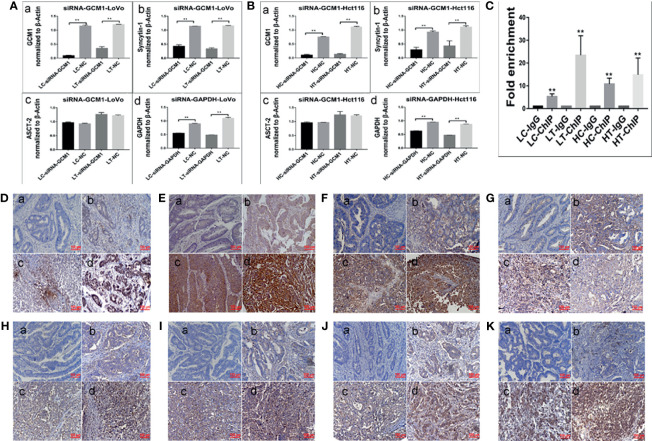
Expression of cell fusion-related proteins and erythroid differentiation-related proteins in human colorectal cancer tissues. **(A)** Column diagram showing the gray value of WB results of total protein expression of GCM1 **(a)**, syncytin-1 **(b)**, ASCT-2 **(c)**, and GADPH **(d)** in LoVo cells with and without ATO treatment after treatment of siRNA-GCM11352, 815, 201, siRNA control, and negative control transfection (^*^P < 0.05,^**^P < 0.01). **(B)** Column diagram showing WB band intensities of total protein expression of GCM1 **(a)**, syncytin-1 **(b)**, ASCT-2 **(c)**, and GADPH **(d)** in Hct116 cells with and without ATO treatment after treatment of siRNA- GCM11352, 815, 201, siRNA control, and negative control transfection. **(C)** Column diagram showing fold enrichment results of ChIP -PCR assay in LoVo and Hct116 cells with and without ATO treatment (^*^P< 0.05,^**^P< 0.01). Expression of erythroid differentiation-related proteins in **(a)** well-differentiated, **(b)** moderately-differentiated, **(c)** poorly-differentiated colorectal cancer tissues, and **(d)** lymph node metastatic tissues. **(D)** GCM1, **(E)** syncytin-1. **(F)** ASCT-2. **(G)** hemoglobin-delta. **(H)** hemoglobin-zeta. **(I)** CD71. **(J)** GATA-1. **(K)** GATA-2. LC, LoVo control cells without ATO treatment; LT, LoVo cells with ATO treatment; HC, Hct116 control without ATO treatment; HT, Hct116 cells with ATO treatment.

GCM1, a placenta-specific transcription factor, has recently been reported to regulate the expression of syncytin gene and consequently trophoblast cell fusion (28). The degradation of GCM1 can be blocked after treatment with the proteasome inhibitor MG132 (29, 30). Our study indicated that the expression of GCM1 in control cancer cells and PGCCs with daughter cells in LoVo and Hct116 cells was increased after MG132 treatment (**Figures 4C, D-a, D-d**). Meanwhile, the expression of syncytin-1 and ASCT-2 was also increased in both control cancer cells and PGCCs cells after MG132 treatment (**Figures 4B, D-b, D-c, D-e, D-f**). Our results also indicated that inhibition of GCM1 by siRNA-GCM1 resulted in a significant reduction in the expression of syncytin-1 (**Figures 4E, F**, **5A, B**). Furthermore, ChIP assay results also demonstrated the interaction between GCM1 and the *syncytin-1* gene in PGCCs with daughter cells after ATO treatment (**Figure 5C**). These results suggested that GCM1 was involved in PGCC formation by regulating *syncytin-1* gene expression. However, there was no change in the expression of ASCT-2 after GCM1 inhibition by siRNA-GCM1.

### Expression of Cell Fusion-Related Proteins and Erythroid Differentiation-Related Proteins in Human Colorectal Cancer Tissues

To assess the expression of cell fusion-related proteins (GCM1, syncytin-1, and ASCT-2) and erythroid differentiation-related proteins (Hemoglobin-delta, Hemoglobin-zeta, CD71, GATA-1, and GATA-2) in human colorectal cancer tissues and their clinicopathological significance, IHC staining was performed on 180 samples of formalin-fixed and paraffin-embedded human colorectal cancer tissues, including well-differentiated (group I), moderately differentiated (group II), poorly differentiated colorectal cancer tissues (group III), and lymph node metastatic tissues (group IV). IHC staining results revealed that the expression of cell fusion-related proteins and erythroid differentiation-related proteins gradually increased from group I to group IV (**Figures 5D–K**). The results of statistical analysis of the expression of cell fusion-related proteins (GCM1, syncytin-1, and ASCT-2) and erythroid differentiation-related proteins (hemoglobin-delta, hemoglobin-zeta, CD71, GATA-1, and GATA-2) were higher in group II than in group I; in group III than in group I; in group IV than in group I; in group III than in group II; in group IV than in group II; and in group IV than in group III (*P* < 0.05, **Table 1**). These results suggested that the expression of cell fusion-related proteins and erythroid cell differentiation-related proteins was closely related to the pathological grade, clinical stage, and distant metastasis of colorectal cancer tissues.

**Table 1:** Comparison of cell fusion and erythroid differentiation-related proteins expression among different groups of human colorectal cancer tissues.

Proteins	Staining Index				*χ^2^*						*P*					
	I(49)	II(42)	III(41)	IV(48)	I *vs* II	I *vs* III	I *vs* IV	II *vs* III	II *vs* IV	III *vs* IV	I *vs* II	I *vs* III	I *vs* IV	II *vs* III	II *vs* IV	III *vs* IV
GCM1	0.80 ± 0.76	1.48 ± 1.04	2.80 ± .94	4.10 ± 2.26	10.10	31.29	49.80	12.03	30.59	7.23	0.001^*^	0.000^*^	0.000^*^	0.001^*^	0.000^*^	0.007^*^
Syncytin-1	1.27 ± .73	2.45 ± .35	4.32 ± .33	5.65 ± .13	18.64	48.90	57.75	14.73	27.11	4.42	0.000^*^	0.000^*^	0.000^*^	0.000^*^	0.000^*^	0.035^*^
ASCT-2	1.65 ± .95	2.81 ± 1.47	5.27 ± .23	7.02 ± .10	15.31	38.62	62.96	11.43	40.66	7.24	0.000^*^	0.000^*^	0.000^*^	0.001^*^	0.000^*^	0.007^*^
HBD	0.73 ± 0.73	1.26 ± 0.80	3.05 ± 1.83	4.21 ± 2.62	9.43	38.03	48.40	23.19	33.53	4.05	0.002^*^	0.000^*^	0.000^*^	0.000^*^	0.000^*^	0.044^*^
HBZ	1.43 ± 0.76	2.62 ± 1.21	4.20 ± 2.24	5.58 ± 3.02	22.86	40.22	55.49	12.15	24.81	4.19	0.000^*^	0.000^*^	0.000^*^	0.000^*^	0.000^*^	0.041^*^
CD71	1.24 ± 1.03	2.45 ± 1.61	6.20 ± 3.31	8.33 ± 3.82	13.12	45.35	66.14	28.13	48.79	6.26	0.000^*^	0.000^*^	0.000^*^	0.000^*^	0.000^*^	0.012^*^
GATA-1	1.49 ± .079	2.43 ± 1.58	4.34 ± 2.59	6.44 ± 3.60	9.37	33.76	47.99	13.07	27.84	7.31	0.002^*^	0.000^*^	0.000^*^	0.000^*^	0.000^*^	0.007^*^
GATT-2	1.10 ± 0.74	2.00 ± 1.41	3.66 ± 2.30	5.04 ± 3.21	9.44	36.42	41.75	10.58	20.62	4.17	0.000^*^	0.000^*^	0.000^*^	0.001^*^	0.000^*^	0.041^*^

Group I, Well-differentiated colorectal cancer tissues; Group II, Moderately-differentiated colorectal cancer tissues; Group III, Poorly-differentiated colorectal cancer tissues; Group IV, Lymph node metastatic tissues; HBD, hemoglobin delta; HBZ, hemoglobin zeta. ^*^P<0.05: statistically significant.

## Discussion

Our recent findings indicate that PGCCs exhibiting cancer stem cell-like properties can be induced from a wide variety of cancer cells using various inducers, including hypoxia, chemotherapy, and radiotherapy, contributing to cellular heterogeneity, stemness, chemoresistance, metastasis, and tumor progression in human solid tumor cells (10, 17, 19, 20). Additionally, PGCCs can form spheroids and generate erythroid cells to form vasculogenic cells, further promoting the progression of cancer. Therefore, PGCCS may be regarded as the evil root of tumor progression, invasion, metastasis, recurrence, heterogeneity, and drug resistance. In the present study, ATO was used to induce PGCC formation in colon cancer cells. PGCCs can generate daughter and erythroid cells *via* asymmetric cell division. Our results also revealed that PGCCs, with their newly generated daughter cells, undergo EMT, indicated by the increased expression of N-cadherin, vimentin, Twist, and Snail and decreased expression of E-cadherin.

PGCCs with daughter cells after ATO treatment can generate erythroid cells with increased expression of embryonic and fetal hemoglobin, including hemoglobin-delta and hemoglobin-zeta, and increased expression of CD71, a fetal cell marker transferrin receptor antigen. Compared to adult hemoglobin, embryonic and fetal hemoglobin exhibit a higher affinity for obtaining oxygen from the surrounding microenvironment to maintain the survival of PGCCs with daughter cells in a hypoxic microenvironment. The generation of PGCC-derived erythroid cells with markedly increased expression of embryonic and fetal hemoglobin further promotes tumor progression by increasing the ability to obtain oxygen (14). In addition, the expression of erythroid differentiation-related proteins, including HIF-1α, EPO, c-Myc, GATA-1, and GATA-2, increases after treatment with ATO. Previously, our results indicated that ectopic expression of c-Myc can promote the generation of nucleated erythroid cells expressing varying levels of embryonic and fetal hemoglobin (14). In the present study, the high expression of HIF-1α and EPO in LoVo and Hct116 cells treated with ATO may indicate the essential role of hypoxia in the generation of PGCC-derived erythroid cells, highly consistent with our previous study findings (10). In addition, the transcription factors GATA1 and GATA2 play crucial roles in gene regulation during hematopoietic differentiation. GATA1 is essential for the differentiation of erythroid cells. GATA2, which is predominantly expressed in hematopoietic stem cells and progenitor cells, regulates their proliferation and maintenance. Overexpression of GATA-2 is common in hematopoietic stem cells and the progenitor population, and this expression can be inhibited concomitantly with an increased expression of GATA1 during terminal erythroid differentiation. This phenomenon is referred to as GATA switching. Therefore, downregulation of GATA-2 and increased expression of GATA-1 are required for erythroid differentiation. However, in our present study, the expression of GATA-1 and GATA-2 increased. Given the reports that increased expression of GATA-2 may cause megakaryopoiesis in hematopoietic progenitors, the expression of CD41, a specific marker of megakaryocytes, was assessed in our study. The expression of CD41 increased, indicating that GATA-2 may also regulate megakaryopoiesis in PGCCs with daughter cells after ATO treatment in addition to erythroid differentiation. Together, our present findings that highly invasive daughter cells and erythroid cells can be generated from PGCCs induced by ATO suggest that PGCCs may be the evil root contributing to the poor effect of ATO on the treatment of solid tumors.

Although the underlying mechanism of PGCC formation is unclear, our previous studies have indicated that syncytin-1 mediated cell fusion may lead to the formation of PGCCs (31, 32). Our present study showed that PGCCs can be formed *via* cell fusion from co-cultured cancer cells stably expressing GFP and RFP after ATO treatment. The PGCCs were yellow when the two images excited from different excitation lights were merged, indicating that the cells expressed both GFP and RFP. Cancer cells expressing GFP fused with cancer cells expressing RFP during the formation of yellow PGCCs. Commonly, eukaryotes have evolved unique mechanisms of cell fusion and nuclear fusion during sexual reproduction. This ensures the formation of a diploid zygote from male and female gametes. Although a similar mechanism of plasma membrane fusion can be observed in the formation of PGCCs between two colon cancer cells, it is unclear whether nuclear fusion is also involved in the formation of PGCCs. Intriguingly, our present results revealed that nuclear fusion can also be observed during the formation of PGCCs from colon cancer cells with stable co-expression of H2B-GFP (green) and H2B-mCherry (red) genes. The nuclear merging of one cancer cell with another cancer cell may be a reasonable explanation of how diploid cancer cells transform into PGCCs with stem cell-like characteristics through cell fusion to promote cancer progression. Cell fusion rapidly alters cellular genotypes and phenotypes and catalyzes genetic diversity by horizontal gene transfer rather than gene mutation (33), especially under various stresses. Cell fusion may contribute to the generation of highly invasive PGCC daughter cells. Cell fusion resembles the fertilization of an egg by a sperm and is similar to the sexual propagation usually used by evolutionarily higher organisms to gain beneficial mutations while purging away deleterious mutations. The resulting hybrid cell receives beneficial genetic material and is usually more malignant; for example, it has a more metastasizing potential.

Despite the role of cell fusion and nuclear fusion in the formation of PGCCs, further research is needed to better understand. One major question concerns the mechanism of the cell fusion pathway. Syncytin-1, which is encoded by human endogenous retrovirus-W, is generally believed to play a crucial role in placental morphogenesis, where it mediates cell-cell fusion of cytotrophoblasts into syncytiotrophoblasts (34, 35). A recent report showed that GCM1 had two GCM1-binding sites in the upstream region of the 5’-LTR of the syncytin-1 gene, activating syncytin-1 gene expression and consequently enhancing syncytin-mediated cell fusion. In addition, syncytin-1-mediated cell fusion is dependent on the interaction with the respective receptor ASCT-2 (36). More recently, it has been reported that the expression of syncytin-1, GCM1, and ASCT-2 is associated with a wide variety of cancers, including colon cancer (37–41). Syncytin-1 mediates not only trophoblast cell fusions but also cancer- and cancer-host cell fusions (37), and ASCT-2 appears to play a role in the aggressiveness of colon cancer (42). A previous study indicated that PGCCs exhibited a developmental pattern similar to that of blastomere-like embryos and exhibited expression of embryonic stem cell markers (20). PGCCs, resembling embryonic blastomeres, can differentiate into all three germ layers *in vitro* (19, 20). The similarities between PGCCs and blastomeres raise the intriguing possibility that PGCCs are somatic equivalents of blastomeres. Although GCM1/syncytin-1 pathway-mediated cell fusion is responsible for the fusion of cytotrophoblasts into the overlying syncytium (25), it is unclear whether the GCM1/syncytin-1 pathway also plays a similar role in the formation of PGCCs. In our study, the expression of cell fusion-related proteins was increased in LoVo and Hct116 cells after treatment with ATO compared to that in control cancer cells without ATO treatment. According to a previous study, the degradation of GCM1 can be blocked by MG132 treatment (29, 30).

## Conclusions

Our study indicated that the expression of GCM1 in control cancer cells and PGCCs with daughter cells in LoVo and Hct116 cells was increased after MG132 treatment. In addition, the expression of syncytin-1 and ASCT-2 was increased in both control cancer cells and PGCCs after MG132 treatment. Our results also indicated that inhibition of GCM1 by siRNA-GCM1 resulted in a significant reduction in the expression of syncytin-1. Furthermore, ChIP assay results also demonstrated the interaction between GCM1 and the *syncytin-1* gene in PGCCs. These results suggest that GCM1 is involved in PGCC formation by regulating *syncytin-1* gene expression. Although the expression of ASCT-2 in both control and PGCC cells was increased after treatment with MG132, its expression after inhibition of GCM1 by siRNA-GCM1 remained unchanged. Together, these results suggest that GCM1/syncytin-1-mediated cell fusion may play a similar role in the development of the placenta and formation of PGCCs. Our understanding of GCM1/syncytin-1-mediated cell fusion in the formation of PGCCs is limited. The possible underlying mechanism of cell fusion in PGCC formation needs to be explored in the future *in vitro and in vivo*. Inspired by the promising effect of ATO in the treatment of APL by targeting CSCs, targeting PGCCs exhibiting CSC-like properties may provide some opportunities for solid tumor therapy.

## Data Availability Statement

The original contributions presented in the study are included in the article/**Supplementary Material**. Further inquiries can be directed to the corresponding author.

## Ethics Statement

The studies involving human participants were reviewed and approved by Tianjin Union Medical Center. Written informed consent for participation was not required for this study in accordance with the national legislation and the institutional requirements.

## Author Contributions

SZ designed the study; collected, analyzed, and interpreted data; contributed to manuscript writing; and approved the manuscript before submission. ZL, MZ, and HZ collected and analyzed data and approved the manuscript before submission. XY, LF, and FF collected, analyzed, and interpreted data, contributed to manuscript writing, and approved the manuscript before submission. JF, RN, and MY collected data, gave constructive comments on the manuscript, and approved the manuscript before submission. All authors contributed to the article and approved the submitted version.

## Funding

This work was supported in part by grants from the National Science Foundation of China (#82173283 and #82103088), and the foundation of committee on science and technology of Tianjin (#20JCYBJC01230). The funders had no roles in the design of the study, data collection, analysis and interpretation, or decision to write and publish the work.

## Conflict of Interest

The authors declare that the research was conducted in the absence of any commercial or financial relationships that could be construed as a potential conflict of interest.

## Publisher’s Note

All claims expressed in this article are solely those of the authors and do not necessarily represent those of their affiliated organizations, or those of the publisher, the editors and the reviewers. Any product that may be evaluated in this article, or claim that may be made by its manufacturer, is not guaranteed or endorsed by the publisher.

## Abbreviations

APL, acute promyelocytic leukemia; ASCT-2, human sodium-dependent neutral amino acid transporter type C-2; ATO, arsenic trioxide; ChIP, chromatin immunoprecipitation; CSC, cancer stem cell; EMT, epithelial to mesenchymal transition; FBS, fetal bovine serum; GCM1, glial cell missing 1; GFP, green fluorescent protein; H&E, hematoxylin and eosin; HIF-1α, hypoxia-inducible factor-1α; ICC, immunocytochemistry; LIC, leukemia-initiating cell; PBS, phosphate-buffered saline; PCR, polymerase chain reaction; PGCC, polyploid giant cancer cell; PML, promyelocytic leukemia; RFP, red fluorescent protein; siRNA, small interfering RNA.
