# Supplementary material for: Arsenic Trioxide Promotes Tumor Progression by Inducing the Formation of PGCCs and Embryonic Hemoglobin in Colon Cancer Cells
Source: Front Oncol. 2021 Oct 5;11:720814. doi: 10.3389/fonc.2021.720814 (PMC8523995; doi:10.3389/fonc.2021.720814)
Supplement: Supplementary file 1 [file DataSheet_1.docx]

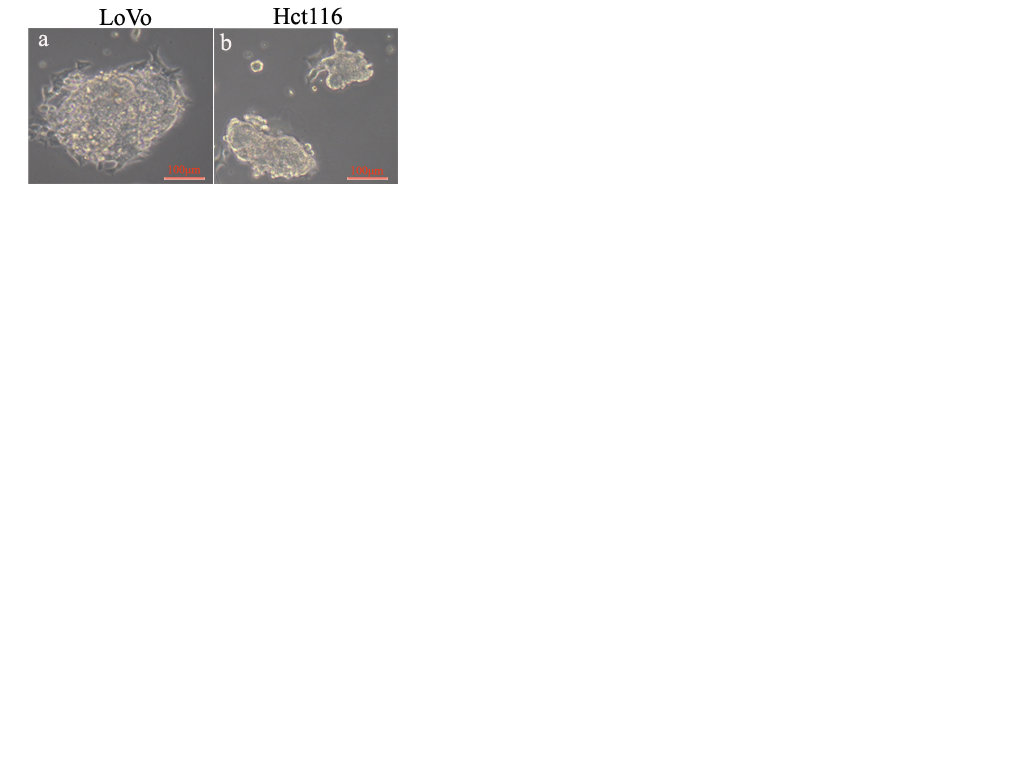


Figure legend: Spheroids formed by ATO treatment. a. Spheroid of LoVo cells treated by ATO (×40). b. Spheroid of Hct116 cells treated by ATO (×40).
