# Supplementary material for: Arsenic Trioxide Promotes Tumor Progression by Inducing the Formation of PGCCs and Embryonic Hemoglobin in Colon Cancer Cells
Source: Front Oncol. 2021 Oct 5;11:720814. doi: 10.3389/fonc.2021.720814 (PMC8523995; doi:10.3389/fonc.2021.720814)
Supplement: Supplementary file 2 [file Table_1.docx]

**Supplementary table 1. Detailed information of the antibodies utilized in this study**

| **Antibody** | **Company (Cat No.)** | **Dilution** |
| --- | --- | --- |
| E-cadherin | Proteintech (20874-1-AP) | 1:50000(WB); 1:500(ICC) |
| Vimentin | Immunoway (YT4879) | 1:2000(WB); 1:200(ICC) |
| Snail | Novus (NBPI-80022) | 1:1000(WB); 1:500(ICC) |
| twist | Proteintech(25465-AP) | 1:1000(WB); 1:500(ICC) |
| Hemoglobin- Delta | Boster(A01076) | 1:2000(WB); 1:400(ICC/IHC) |
| Hemoglobin-Zeta | Fitzgerald(70R-1234) | 1.25μg/ml(WB); 8μg/ml(ICC/IHC) |
| CD71 | Proteintech(10084-2-AP) | 1:2000(WB); 1:400(ICC/IHC) |
| HIF-1α | Abcam(Ab51608) | 1:1000(WB); 1:500(ICC) |
| EPO | Proteintech(66975-1-lg) | 1:50000(WB); 1:500(ICC） |
| c-Myc | Proteintech(10828-1-AP) | 1:4000(WB); 1:500(ICC) |
| GATA-1 | Proteintech(10917-2-AP) | 1:2000(WB); 1:200(ICC/IHC) |
| GATA-2 | Proteintech(11103-1-AP) | 1:3000(WB); 1:200(ICC/IHC) |
| CD41 | Proteintech(24552-1-AP) | 1:5000(WB);1:200(ICC) |
| ERK1/2 | Proteintech(16443-1-AP) | 1:4000(WB); 1:500(ICC) |
| P-Erk1/2(rabbit) | CST(mAb #4370) | 1:2000(WB) |
| P-Erk1/2(mouse) | CST(mAb #5726) | 1:1000(WB) |
| P38 MAPK | Proteintech(14064-1-AP) | 1:2000(WB); 1:200(ICC) |
| β-catenin | Proteintech(17565-1-AP) | 1:10000(WB); 1:500(ICC) |
| GCM1 | Abnova(H00008521-M05) | 4μg/m(WB); 1.7μg/ml(ICC/IHC) |
| GCM1 | Santa Cruz(SC-101173) | 20μl for ChIP |
| syncytin-1 | Bioboss(bs-2962R) | 1:2000(WB); 1:1000(ICC/IHC) |
| ASCT-2 | Proteintech(20350-1-AP) | 1:10000(WB); 1:1000(ICC/IHC) |
| GAPDH | Zhongshan, Beijing（TA-08） | 1:1000(WB) |
| β-actin | Zhongshan, Beijing（TA-09） | 1:1000(WB) |
| Anti-rabbit IgG | Cell Signaling Technology（7074S） | 1:3000(WB) |
| Anti-mouse IgG | Cell Signaling Technology（7076S） | 1:3000(WB) |

WB: Western blot；ICC:Immunocytochemistry staining；IHC: Immunohistochemical staining

ChIP:Chromatin immunoprecipitation
